# Chinese highly pathogenic porcine reproductive and respiratory syndrome virus exhibits more extensive tissue tropism for pigs

**DOI:** 10.1186/1743-422X-9-203

**Published:** 2012-09-17

**Authors:** Limin Li, Qian Zhao, Xinna Ge, Kedao Teng, Yu Kuang, Yanhong Chen, Xin Guo, Hanchun Yang

**Affiliations:** 1 Key Laboratory of Animal Epidemiology and Zoonosis of Ministry of Agriculture, College of Veterinary Medicine and State Key Laboratory of Agrobiotechnology, China Agricultural University, No. 2 Yuanmingyuan West Road, Haidian District, Beijing, 100193, China

**Keywords:** Porcine reproductive and respiratory syndrome virus, High pathogenicity, Tissue tropism, Immunohistochemistry

## Abstract

**Background:**

The highly pathogenic porcine reproductive and respiratory syndrome virus (PRRSV) emerging in China exhibits high fatality to pigs. However, the mechanism related to the increased pathogenicity of the virus remains unclear. In the present study, the differences in tissue tropism between the highly pathogenic PRRSV strain (JXwn06) and the low pathogenic PRRSV strain (HB-1/3.9) were investigated using PRRSV-specific immunohistochemistry (IHC) staining to provide evidence for elucidating possible mechanism of the pathogenicity of Chinese highly pathogenic PRRSV.

**Findings:**

IHC examination showed that PRRSV antigen in the tissues including spleen, tonsil, thymus, kidney, cerebellum, stomach, small intestine, large intestine, turbinal bone and laryngeal cartilage was positive in more pigs inoculated with JXwn06 than HB-1/3.9, and the tissues including trachea, esophagus, liver, mandibular gland and thyroid gland were positive for viral antigen in the pigs inoculated with JXwn06, but not in the pigs inoculated with HB-1/3.9. Meanwhile, we observed that epithelium in tissues including interlobular bile duct in liver, distal renal tubule of kidney, esophageal gland and tracheal gland were positive for viral antigen only in JXwn06-inoculated pigs, and epithelium of gastric mucosa and fundic gland, and intestinal gland were positive for viral antigen in both JXwn06- and HB-1/3.9-inoculated pigs, using monoclonal antibodies to N and Nsp2 proteins.

**Conclusions:**

Taken together, these findings indicate that the highly pathogenic PRRSV JXwn06 displays an expanded tissue tropism in *vivo*, suggesting this may contribute to its high pathogenicity to pigs.

## Findings

Porcine reproductive and respiratory syndrome (PRRS) has become a well-recognized global swine disease [1,2] since it was first reported as a “mystery swine disease” in the United States in 1987 [3]. The disease is predominantly characterized by reproductive failure in sows, pre-weaning mortality, and respiratory disorders in pigs of all ages [4,5], resulting in great economic losses for the swine industry [6].

Porcine reproductive and respiratory syndrome virus (PRRSV), the causative agent of the disease, is a member of the family *Arteriviridae*[7]. Previous studies have indicated that PRRSV infects macrophages in some tissues of pigs and mainly replicates within macrophages in the respiratory (lung) and lymphoid systems [8,9]. Swine pulmonary alveolar macrophages are the predominantly cells for virus replication [10,11] although immature sperm cells and epithelial pulmonary cells were recognized to also be infected by PRRSV [12]. A number of investigations revealed that the largest amount of viral antigen and/or nucleic acid could be observed in lungs and lymph nodes of the pigs following PRRSV infection [10,12-15]. Therefore, PRRSV mainly has a tropism for the respiratory and lymphoid tissues of pigs. In addition, it has been shown that the tissue tropism, the cell types in which PRRSV antigen was detected and distribution of PRRSV antigen-positive cells within particular tissues and organs are generally similar among PRRSV strains with differences in virulence, even though the isolates with increased virulence often replicate better in vivo than the less virulent isolates [14,16].

Since 2006, the clinical outbreaks of atypical PRRS caused by a highly pathogenic PRRSV with unique 30-amino-acid (30-aa) deletion in its nonstructural protein 2 (Nsp2)-coding region were widespread in major pig-producing areas of China [17]. These outbreaks have brought great economic losses to the swine industry in China due to the high mortality and morbidity in the affected pig herds. To date, all the evidences have pointed to the conclusion that the highly pathogenic PRRSV with the 30-aa deletion in Nsp2-coding region is the causative agent of atypical PRRS in China, and the virus has marked characteristic of high pathogenicity aside from the genomic characteristic of 30-aa deletion in Nsp2, compared with previous Chinese strains [18-20]. Although our recent findings have revealed that the 30-aa deletion in Nsp2 is not related to its virulence [20], the mechanisms associated with high pathogenicity of the virus for pigs remain unclear.

In the present study, we observed the distribution of viral antigen in tissues of the pigs experimentally infected with a highly pathogenic PRRSV strain (JXwn06) and simultaneously analyzed the tissue tropism of the virus in comparison with a low pathogenic strain (HB-1/3.9), in order to provide valuable information that might be helpful for explaining differences in the virulence of these viruses.

Two PRRSV strains that differ in virulence, JXwn06 and HB-1/3.9, were used for the study. Starin JXwn06, a highly pathogenic strain that cause fatality for pigs, was isolated from an intensive pig farm with an atypical PRRS outbreak in the Jiangxi province of China in 2006 [20]. HB-1/3.9, a low-virulence and adapted strain in MARC-145 cells that induce no fatality for pigs, was derived from HB-1(sh)/2002 isolated in 2002 [21]. The genomic sequence of JXwn06 shared 97.4% nucleotide identity with HB-1/3.9. The marked genomic difference between the two viruses is that a 30-aa deletion exists in Nsp2-coding region of JXwn06, not in HB-1/3.9 [20]. Thirteen 6-week-old specific-pathogen free (SPF) Landrace piglets, free of PRRSV, porcine circovirus type 2, classical swine fever virus, porcine parvovirus, pseudorabies virus, swine influenza virus and *Mycoplasma hyopneumoniae*, were obtained from the Beijing Center for SPF Swine Breeding and Management. The animals were allocated randomly to three groups—JXwn06-inoculated group (n = 5), HB-1/3.9-inoculated group (n = 5) and control group (n = 3). Each group was housed separately in a different isolation room, with individual ventilation. Each piglet in the JXwn06-inoculated group or HB-1/3.9-inoculated group was administered intranasally at a dose of 2 × 10^5.0^ TCID_50_ of the fifth-passage viral cultures on MARC-145 cells. The piglets in the control group were exposed in the same manner to uninfected MARC-145 cell culture supernatant. Following the inoculations, all animals were clinically observed and rectal temperatures were recorded daily until death or necropsy. Serum samples were collected prior to inoculation and from day 1 onwards post-inoculation (PI) for virus detection by RT-PCR using the primers (the forward primer: 5’-CAAATAACAACGGCAAGCAG-3’; the reverse primer: 5’-AAACTCCACAGTGTAACTTAT-3’) to amplify ORF7 gene of PRRSV. Tissues of JXwn06-inoculated pigs were collected when the pigs died. HB-1/3.9-inoculated pigs were euthanized for necropsy on day 7 PI when the peak of viremia occurred during a three-week-experimental period in our previous study [20], and their tissues were collected. Also, on day 7 PI, the control pigs were euthanized for necropsy and their tissues were collected. Total collected tissues are listed in Table 1. All the tissues were fixed in 4% neutral buffered polyoxymethylene for immunohistochemistry (IHC) examination of viral antigen. The experimental researches on animals in this study have followed international recognized guidelines, and have been approved by The Beijing Municipal Committee of Laboratory Animal Management and The Ethics Committee of China Agricultural University.

**Table 1:** Distribution and staining intensity of PRRSV antigen-positive tissues by IHC using monoclonal antibody to N protein

**Tissues**	**JXwn06-inoculated pigs**	**HB-1/3.9-inoculated pigs**
	**Number of positive or negative**	**Number of positive or negative**
Lung	+ 5/5	+ 5/5
Trachea	+ 5/5	- 5/5
Turbinal bone	+ 5/5	+ 3/5
Spleen	+ 5/5	+ 3/5
Tonsil	++ 5/5	+ 4/5
Thymus	+ 3/5	+ 1/5
Mesenteric lymph node	+ 5/5	+ 5/5
Inguinal lymph node	+ 5/5	+ 5/5
Laryngeal cartilage	+ 5/5	+ 2/5
Esophagus	++ 5/5	- 5/5
Stomach	++ 5/5	+ 1/5
Small intestine	++ 5/5	++ 1/5
Large intestine	++ 5/5	++ 1/5
Liver	+ 5/5	- 5/5
Cerebrum	+ 4/5	- 5/5
Cerebellum	+ 3/5	+ 1/5
Kidney	++ 5/5	+ 1/5
Mandibular gland	++ 5/5	- 5/5
Thyroid gland	++ 5/5	- 5/5
Heart	- 5/5	- 5/5

Notes: All the tissue sections were observed under 400× in magnification, and the average positive cells in five visual fields randomly of each section were counted. + represents < 10 positive cells; ++ means > 10 positive cells; - denotes no positive cell.

**Figure 1:**
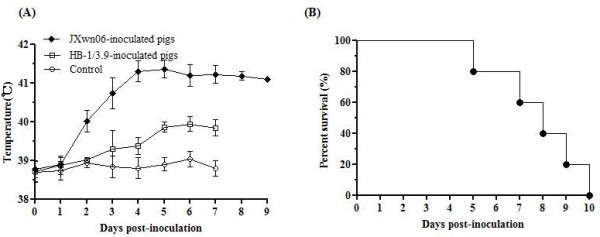
** Rectal temperature and the survival curve of the inoculated pigs.** Shown are rectal temperature change (**A**) and survival rates (**B**) of the JXwn06-inoculated pigs. The data for body temperature are means ± standard deviations (error bars), except for the temperature of the one JXwn06-inoculated pig that survived on day 9 PI.

From day 2 PI onwards, the body temperatures of pigs in JXwn06-inoculated group started to rise and had a mean peak of 41.3°C on day 4 PI, while the pigs in HB-1/3.9-inoculated group showed slightly an elevated body temperature from day 3 PI onwards (Figures 1A). The body temperatures of pigs inoculated with JXwn06 were significantly higher than those of pigs inoculated with HB-1/3.9 from on day 3 PI onwards (*p* < 0.01). The pigs inoculated with JXwn06 showed marked clinical signs, including depression, anorexia and lethargy, rubefaction on skin and in ears, respiratory distress, shivering and diarrhea, and died within 5–10 days PI (Figures 1B), whereas the pigs inoculated with HB-1/3.9 presented no obvious clinical manifestations within 7 days PI. All the serum samples from both JXwn06- and HB-1/3.9-inoculated pigs on days 3 to 7 PI were positive for viral nucleic acid detection by RT-PCR (Table 2). It is worthy to note that the virus could be detected earlier on day 1 PI, in the group inoculated with the highly pathogenic JXwn06. Our previous data also showed the virus titers in the serum of the pigs infected with JXwn06 were much higher than those of the pigs infected with HB-1/3.9 [20]. For the control pigs, no body temperature elevation or clinical signs were observed, and no virus was detected in serum samples.

**Table 2:** Viremia detection of the inoculated pigs by RT-PCR

**DPI**	**JXwn06-inoculated pigs**	**HB-1/3.9-inoculated pigs**
0	−5/5^a^	−5/5
1	+ 5/5	−5/5
2	+ 5/5	- 5/5
3	+ 5/5	+ 4/5
4	+ 5/5	+ 5/5
5	+ 4/4	+ 5/5
6	+ 4/4	+ 5/5
7	+ 3/3	+ 5/5
8	+ 3/3	NA^b^
9	+ 1/1	NA
10	NA	NA

^a^ positive or negative number / animal number, + positive, - negative.

^b^ not applicable.

**Figure 2:**
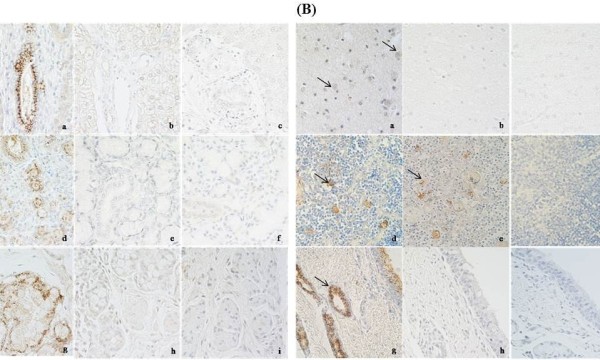
** IHC staining of some tissues by using monoclonal antibody to N protein of PRRSV.** (**A**): a, d, g- liver, mandibular gland and Esophagus of JXwn06-inoculated pigs, respectively (positive); b, e, h- liver, mandibular gland and Esophagus of of HB-1/3.9-inoculated pigs, respectively (negative);c, f, i- liver, mandibular gland and Esophagus of control pigs, respectively. (**B**): a, d, g- cerebrum, thymus and trachea of JXwn06-inoculated pigs, respectively; b, e, h- cerebrum, thymus and trachea of HB-1/3.9-inoculated pigs, respectively; c, f, i- cerebrum, thymus and trachea of control pigs, respectively; arrows point to the positive signals. The tissue sections were observed under 400× magnification.

The monoclonal antibody against PRRSV N protein (diluted 1:100) [22] was used for the detection of PRRSV antigen in the tissues of the inoculated pigs. The number and intensity of PRRSV antigen-positive tissues between JXwn06-inoculated pigs and HB-1/3.9-inoculated pigs are summarized in Table 1. The results showed that PRRSV antigen was detected in trachea, esophagus, liver, cerebrum, mandibular gland and thyroid gland in JXwn06-inoculated pigs; while, no positive signals were found in these tissues in HB-1/3.9-inoculated pigs. The positive tissues including liver, mandibular gland, cerebrum, and esophagus of JXwn06-inoculated pigs are shown in Figures 2. Lung, turbinal bone, laryngeal cartilage, stomach, intestine, cerebellum, kidney, spleen, tonsil, thymus and lymph node were the tissues in which positive signals were detected both in JXwn06-inoculated pigs and HB-1/3.9-inoculated pigs. It is noteworthy that the positive rate of PRRSV antigen in JXwn06-inoculated pigs was higher than in HB-1/3.9-inoculated pigs for turbinal bone (5/5 vs. 3/5), laryngeal cartilage (5/5 vs. 2/5), small intestine (5/5 vs. 1/5), large intestine (5/5 vs. 1/5), cerebellum (3/5 vs. 1/5), spleen (5/5 vs. 3/5) and thymus (3/5 vs. 1/5); and more positive cells were observed in stomach, kidney and tonsil of JXwn06-inoculated pigs compared to HB-1/3.9-inoculated pigs.

PRRSV antigen-positive cells observation showed that both the highly and low pathogenic virus has a tropism for macrophages in the respiratory and lymphoid systems of the inoculated pigs. Lymph node, tonsil, spleen and thymus were the tissues with positive signals mainly in macrophages which were identified both in JXwn06- and HB-1/3.9-inoculated pigs, and the positive cells were mostly distributed in medullar substance and paracortical area in lymph organs (Additional file 1). In addition, strong positive signals could be observed in epithelium of some of tissues of both JXwn06- and HB-1/3.9-inoculated pigs. Besides pulmonary alveolar macrophages, bronchial epithelium was positive in lung of the inoculated pigs. In stomach, small intestine, large intestine, laryngeal cartilage and turbinal bone, viral antigen was mainly seen in the mucous membrane epithelium or glandular epithelium. The epithelium of interlobular bile duct in liver, distal renal tubule in kidney, esophageal gland and tracheal gland were found positive only in JXwn06-inoculated pigs. These findings indicate that the highly pathogenic PRRSV JXwn06 exhibits an expanded tissue tropism in *vivo* compared to the low pathogenic PRRSV HB-1/3.9, suggesting that JXwn06 has an increased ability to replicate in *vivo* compared to HB-1/3.9. In addition, the hearts were negative for viral antigen in both JXwn06- and HB-1/3.9-inoculated pigs, this contradicts with earlier reports that macrophages and endothelial cells in heart could be infected by PRRSV [8,10], suggesting this might be due to the pathogenicity differences among the virus strains. No positive signals were observed in any tissues from the control pigs or when PBS or normal mouse sera were used as a substitution for the primary antibody for IHC staining.

**Figure 3:**
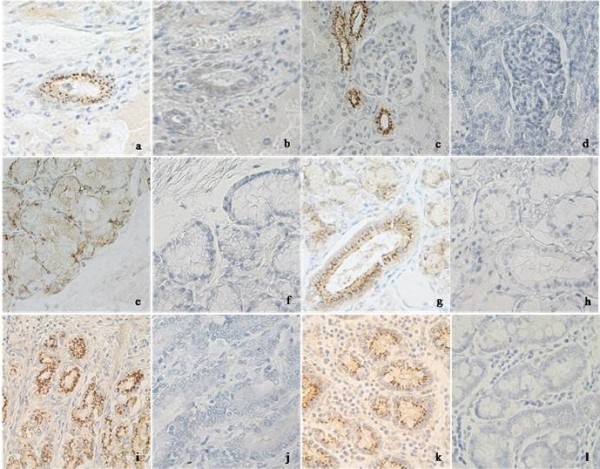
** IHC staining of epithelium in tissues by using monoclonal antibody to Nsp2 of PRRSV.** a, c, e, g, i, k **-** the epithelium of interlobular bile duct in liver, distal renal tubule in kidney, esophageal gland and mandibular gland, the epithelium of gastric fundic gland and intestinal gland from JXwn06- inoculated pigs, respectively; b, d, f, h, j, l - the corresponding tissues stained by normal mice sera as primary antibody. The tissue sections were observed under 400× magnification.

PRRSV antigen-positive tissues detected with monoclonal antibody against N protein were further stained using monoclonal antibody against Nsp2 (diluted 1:400) [23]. Positive signals detected using the two antibodies were consistent. The results showed that the positive signals were observed not only in macrophages mainly in lymphoid organs, but also in epithelium including esophageal gland, gastric mucous membrane and fundic gland, intestinal gland, interlobular bile duct in liver and mandibular gland as well as renal tubule in kidney. It could be speculated that this may be due to the accumulation of viral particles within the epithelium of these tissues or the consequence resulting from the replication of PRRSV in epithelial cells within these tissues. However, the latter needs to be further confirmed in vitro. Partial tissues with positive signals in epithelium are shown in Figures 3.

Our present findings describe the tissue distribution of viral antigen of a Chinese highly pathogenic strain of PRRSV using IHC. In summary, the highly pathogenic PRRSV emerging in China exhibits an expanded tissue tropism in *vivo*, suggesting a possible mechanism that contributes to its high pathogenicity for pigs.

## Competing interests

The authors declare that they have no competing interests.

## Authors’ contributions

LML carried out animal experiment, performed IHC staining of the tissues and wrote the manuscript. QZ and YHC participated in animal experiment. XNG conducted RT-PCR for PRRSV detection. KDT and YK participated in the preparation of tissue sections and IHC staining. XG and HCY participated in study design and coordination, and revised the manuscript. All authors approved the final manuscript.

## Supplementary Material

Additional file 1 IHC staining of lung and lymph node of the inoculated pigs using monoclonal antibody to N protein. Click here for file
