# Supplementary material for: Chinese highly pathogenic porcine reproductive and respiratory syndrome virus exhibits more extensive tissue tropism for pigs
Source: Virol J. 2012 Sep 17;9:203. doi: 10.1186/1743-422X-9-203 (PMC3487961; doi:10.1186/1743-422X-9-203)

Additional file

IHC staining pictures of lung and lymph node of the inoculated pigs using monoclonal antibody to N protein

Lung and lymph node tissue sections of the JXwn06- and HB-1/3.9-inoculated pigs were stained by IHC using monoclonal antibody to N protein of PRRSV, and observed under 400× magnification. a, lung section of HB-1/3.9-inoculated pig; b, lung section of JXwn06-inoculated pig; c, lung section of control pig; d, lymph node section of HB-1/3.9-inoculated pig; e, lymph node section of JXwn06-inoculated pig; f, lymph node section of control pig.

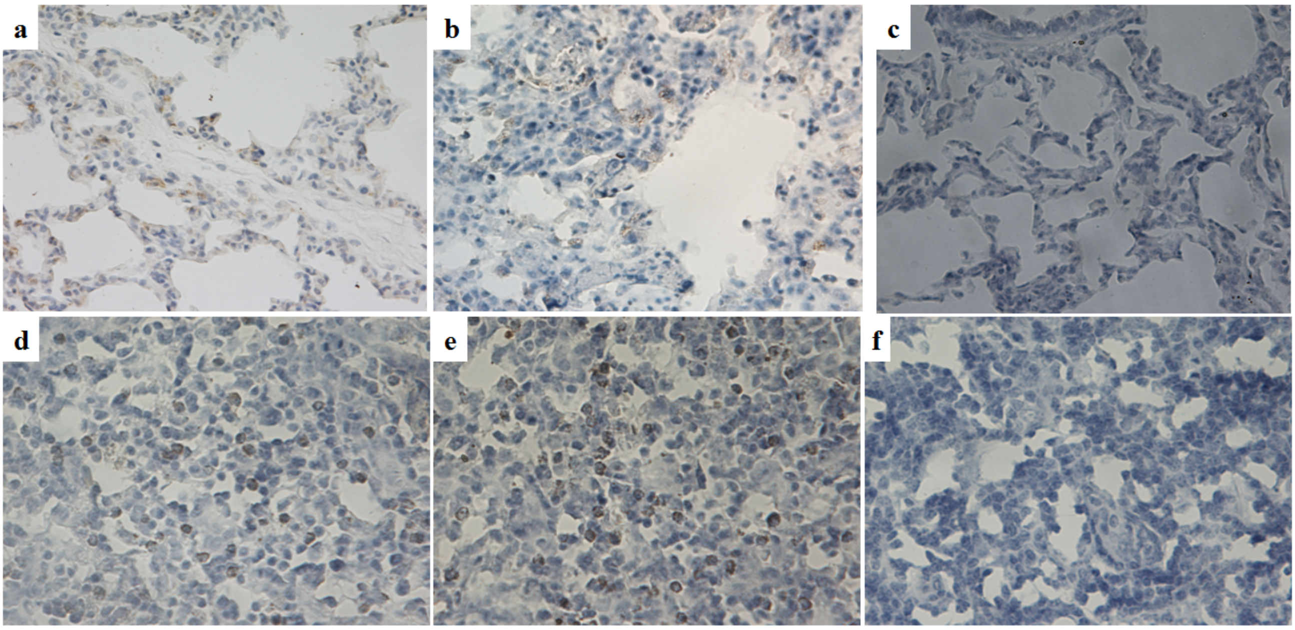

Supplement: Additional file 1 — IHC staining of lung and lymph node of the inoculated pigs using monoclonal antibody to N protein. [file 1743-422X-9-203-S1.pdf]
